# Dissimilarity of the gut–lung axis and dysbiosis of the lower airways in ventilated preterm infants

**DOI:** 10.1183/13993003.01909-2019

**Published:** 2020-05-07

**Authors:** David Gallacher, Emma Mitchell, Dagmar Alber, Richard Wach, Nigel Klein, Julian R. Marchesi, Sailesh Kotecha

**Affiliations:** 1 Dept of Child Health, School of Medicine, Cardiff University, Cardiff, UK; 2 Institute of Child Health, University College London, London, UK; 3 Neonatal Unit, North Bristol NHS Trust, Bristol, UK; 4 School of Biosciences, Cardiff University, Cardiff, UK; 5 Division of Integrative Systems Medicine and Digestive Disease, Imperial College London, London, UK; 6 Joint first authors

## Abstract

**Background:**

Chronic lung disease of prematurity (CLD), also called bronchopulmonary dysplasia, is a major consequence of preterm birth, but the role of the microbiome in its development remains unclear. Therefore, we assessed the progression of the bacterial community in ventilated preterm infants over time in the upper and lower airways, and assessed the gut–lung axis by comparing bacterial communities in the upper and lower airways with stool findings. Finally, we assessed whether the bacterial communities were associated with lung inflammation to suggest dysbiosis.

**Methods:**

We serially sampled multiple anatomical sites including the upper airway (nasopharyngeal aspirates), lower airways (tracheal aspirate fluid and bronchoalveolar lavage fluid) and the gut (stool) of ventilated preterm-born infants. Bacterial DNA load was measured in all samples and sequenced using the V3–V4 region of the 16S rRNA gene.

**Results:**

From 1102 (539 nasopharyngeal aspirates, 276 tracheal aspirate fluid, 89 bronchoalveolar lavage, 198 stool) samples from 55 preterm infants, 352 (32%) amplified suitably for 16S RNA gene sequencing. Bacterial load was low at birth and quickly increased with time, but was associated with predominant operational taxonomic units (OTUs) in all sample types. There was dissimilarity in bacterial communities between the upper and lower airways and the gut, with a separate dysbiotic inflammatory process occurring in the lower airways of infants. Individual OTUs were associated with increased inflammatory markers.

**Conclusions:**

Taken together, these findings suggest that targeted treatment of the predominant organisms, including those not routinely treated, such as *Ureaplasma* spp., may decrease the development of CLD in preterm-born infants.

## Abstract 2

**Respiratory colonisation was acquired after birth and associated with a pro-inflammatory response, suggesting an infectious process was present in babies at risk of developing chronic lung disease of prematurity (CLD), thus providing a target to reduce CLD** http://bit.ly/31C27iX

## Introduction

Chronic lung disease of prematurity (CLD), also called bronchopulmonary dysplasia, is a major consequence of preterm birth being influenced by many early life factors, including delivery at an early stage of lung development, need for respiratory support and supplemental oxygen therapy [1]. We have previously shown that the presence of predominant bacteria is associated with the development of CLD, probably *via* the pulmonary inflammatory pathway [2, 3]. Furthermore, specific organisms such as *Ureaplasma* are strongly implicated in the pathogenesis of CLD [4, 5], with some evidence suggesting that eradication of *Ureaplasma* may decrease rates of CLD [6]. In the term-born infant, early bacterial colonisation of the upper respiratory tract appears to occur within minutes of birth [7] or possibly antenatally, but the natural history of colonisation of the upper and lower airways of the preterm lung is less clear [8]. A recent systematic review [9] investigating the development of the airway microbiome in preterm infants raised more questions about the bacterial colonisation of the preterm lung than have been answered with existing studies; no study has investigated the links between the upper airways and the gastrointestinal tract, *i.e.* the proposed gut–lung axis, with the lower airways of preterm infants. The six studies included [10–15] showed variable findings, *e.g.* one reported progression from Proteobacteria to Firmicutes, but another showed reverse progression. To provide a more comprehensive assessment of the bacterial community in preterm infants who are at risk of developing CLD, we 1) serially studied multiple anatomical sites, including obtaining tracheal aspirate fluid (TAF) and bronchoalveolar lavage (BAL) fluid from ventilated preterm infants to assess the tracheal and lower airways, respectively; and nasopharyngeal aspirates (NPA) and stool samples to assess the progression of the bacterial community in ventilated preterm infants over time in the upper and lower airways; 2) assessed the microbial relationship between the stool and upper/lower airways, *i.e.* the gut–lung axis, for which there are no studies thus far in term or preterm-born infants; 3) assessed the relationship between the bacterial communities and clinical factors including mode of delivery, sex, clinical site of sampling and effect of antibiotics; and 4) measured proinflammatory cytokines interleukin (IL)-6 and -8 in all sample types and related the findings to the bacterial communities observed.

## Methods

### Sample collection

Preterm infants of ≤32 weeks' gestation were recruited from two tertiary regional neonatal intensive care units in the United Kingdom. TAF and NPA were collected at the time of routine endotracheal tube (ETT) suctioning. BAL samples were collected as per our previous studies [2, 16] and according to the European Respiratory Society task force guidelines [17] at the time of routine ETT suctioning, as described in the supplementary material. TAF, BAL and NPA samples were collected daily during the first week then twice weekly or until extubation, whichever occurred earlier. Stool samples were collected weekly until 28 days of life. Ethical approval (ref 14/WA/0190) was obtained from the Wales Research Ethics Committee 2 and written informed consent was obtained from the parents.

### DNA extraction, bacterial load and sequencing

DNA extraction, bacterial load and 16S sequencing are described in more detail in the supplementary material. Total bacterial load was quantified using a quantitative PCR assay based on published protocols [18] amplifying the V3–V4 region of the 16S rRNA gene. Purified PCR products were quantified and samples with adequate amplification pooled to create an amplicon library. Separate libraries were prepared for TAF, BAL, NPA and stool samples. Each library was sequenced on an Illumina MiSeq (San Diego, CA, USA) device [19].

### Data analysis

Mothur v1.39.5 [20] was used to process the sequencing data, excluding chimeric sequences and low-quality reads and to group the DNA sequence data into operational taxonomic units (OTUs) at 3% dissimilarity. Samples with <1000 reads were excluded. The reference database from the Ribosomal Database Project [21] was used for phylogenic identification of each OTU from kingdom to genus level. Samples were rarefied to 1000 reads per sample prior to further analysis. Statistical analysis was performed within R v3.4.2 (www.r-project.org) using Phyloseq v1.20.0 [22] and Vegan (https://cran.r-project.org/web/packages/vegan/index.html). STAMP v2.13 [23] was used to compare bacterial profiles between different groups. Datasets were merged at genus level for comparison of microbiome profiles from different anatomical sites. Samples with a single OTU representing >50% reads were considered to contain a predominant organism.

## Results

1102 samples were collected from 55 preterm infants born at ≤32 weeks' gestation (median 26.0 weeks, interquartile range 24.7–27.5 weeks) during the first 28 days of age including 539 NPA, 276 TAF, 89 BAL and 198 stool samples. The demographics are shown in table 1, including comparison of data between the two recruitment centres (University Hospital of Wales and North Bristol Trust (NBT)). Marginally more immature infants were recruited at NBT, although the difference was not statistically significant (26.8 *versus* 25.9 weeks, p=0.07). More patent ductus arteriosus were identified at NBT, since they screen for this condition. As expected with recruitment of only ventilated preterm infants, most infants developed CLD defined using the National Institute of Child Health and Human Development definitions [24], with 84% developing moderate to severe CLD.

**TABLE 1:** Demographics of recruited preterm infants from two sites

	**All infants**	**Infants recruited from UHW**	**Infants recruited from NBT**	**p-value (UHW *versus* NBT)**
**Infants**	55	20 (36)	35 (64)	
**Samples**				
Nasopharyngeal aspirates	539	145	394	
Tracheal aspirate fluid	276	62	214	
Bronchoalveolar lavage fluid	89	89	0	
Stool	198	64	134	
Total	1102	360	742	
**Male**	36 (65)	12 (60)	24 (69)	0.73
**Gestation weeks**	26.0 (24.7–27.5)	26.8 (25.3–29.4)	25.9 (24.7–26.6)	0.07
**Birthweight g**	764 (680–918)	835 (695–1082)	746 (677–880)	0.16
**Antenatal steroids**	51/55 (93)	18/20 (90)	33/35 (94)	0.18
**Vaginal delivery**	29 (53)	10 (50)	19 (54)	0.98
**Multiple births**	17 (31)	6 (30)	11 (31)	1
**Maternal antibiotics in labour**	14 (25)	4 (20)	10 (29)	0.70
**Surfactant administration**	55/55 (100)	20/20 (100)	35/35 (100)	0.27
**CLD severity^#^**				
None	5 (9)	4 (20)	1 (3)	0.02
Mild	4 (7)	3 (15)	1 (3)	
Moderate	18 (33)	3 (15)	15 (43)	
Severe/died	28 (51)	10 (50)	18 (51)	
**Survival to discharge**	47 (85)	17 (85)	30 (86)	1
**Ventilation days**	17 (4–32.5)	25 (3.5–37.8)	17 (5–28.5)	0.55
**Noninvasive ventilation days**	47 (24.5–64.5)	38 (19.8–53.5)	55 (31.5–66)	0.14
**Length of hospital stay days**	93 (69.5–130)	104.5 (52.8–136.5)	93 (80–122)	0.95
**Necrotising enterocolitis^¶^**				
Grade 1	7 (13)	4 (20)	3 (9)	1
Grade 2	1 (2)	0 (0)	1 (3)	
Grade 3	6 (11)	1 (5)	5 (14)	
**Patent ductus arteriosus**	40 (73)	11 (55)	29 (83)	0.06
**Initial breast milk**	55 (100)	20 (100)	35 (100)	1
**Discharge breast milk**	17 (31)	6 (35)	11 (37)	1

Data are presented as n, n (%) or median (interquartile range), unless otherwise stated. UHW: University Hospital Wales; NBT: North Bristol Trust; CLD: chronic lung disease of prematurity. ^#^: categorised according to [24] ; ^¶^: categorised according to Bell's staging criteria.

**FIGURE 1:**
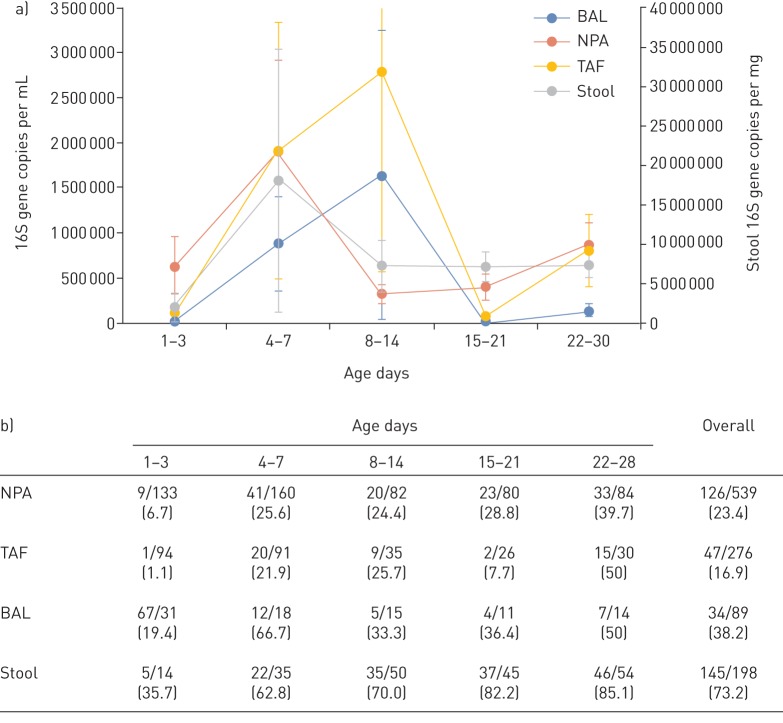
Total bacterial load in the gut and airways of preterm infants. a) Change in total bacterial load over time shown for the four anatomical sites: bronchoalveolar lavage (BAL), nasopharyngeal aspirate (NPA), tracheal aspirate fluid (TAF) and stool samples. BAL, TAF and NPA samples were measured in copies of the 16S RNA gene per mL of supernatant. Stool samples were measured in copies of the 16S DNA gene per mg of stool. Data are presented as mean±se. b) Table showing the number of samples available at each time point for each sample type, and the percentage of the samples from which the 16S rRNA gene could be successfully amplified at each time point of each sample type. Data are presented as n/N (%).

352 (32%) of the 1102 samples were successfully sequenced for the 16S rRNA gene with the greatest success rate for stool (73%) samples and least for TAF (17%) samples (figure 1). Bacterial load was low at days 1–3, with only 1.1% and 6.7% of TAF and NPA samples, respectively, successfully sequenced for the bacterial 16S rRNA gene. We obtained a success rate of 35.7% for stool samples, suggesting a low bacterial load at birth, especially in the upper airways. Although 19.4% of BAL samples were available for DNA sequencing, a lower threshold for bacterial DNA sequencing was used for these samples (concentration of DNA post-PCR amplification of 0.3 ng·µL^−1^ for TAF and NPA, 0.1 ng·µL^−1^ for BAL and 0.5 ng·µL^−1^ for stool). Bacterial load peaked at day 4–7 for BAL and TAF samples and at days 8–14 for NPA and stool samples. Interestingly, a similar pattern of increase was observed for the TAF and BAL samples as for the stool and NPA samples (figure 1).

After low-quality reads and chimeric sequences were removed, the mean (range) reads were 109 457 (8665–426 696) for TAF samples; 44 548 (27–177 875) for BAL samples; 60 295 (5951–222 539) for NPA samples; and 59 329 (1–481 757) for stool samples, with 0, 2, 0 and 39 samples removed, respectively, for reads of <1000 per sample. The maximum reads for negative controls were 7558, 588, 580 and 206, respectively, with one TAF sample discarded due to co-clustering with a negative control sample.

**FIGURE 2:**
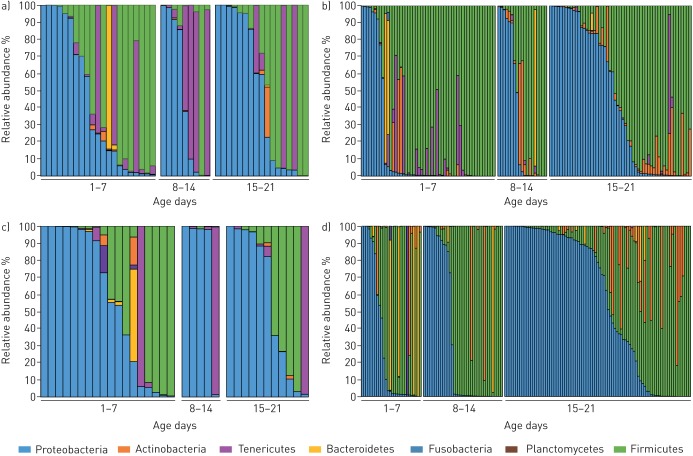
Microbiome analyses for each sample site shown by phylum: a) tracheal aspirate fluid; b) bronchoalveolar lavage; c) nasopharyngeal aspirate; and d) stool. The relative abundance of the predominant phyla of the preterm infant is shown for each time period. Each bar represents an individual sample with relative abundance shown after all operational taxonomic units from each phylum were combined and averaged.

**FIGURE 3:**
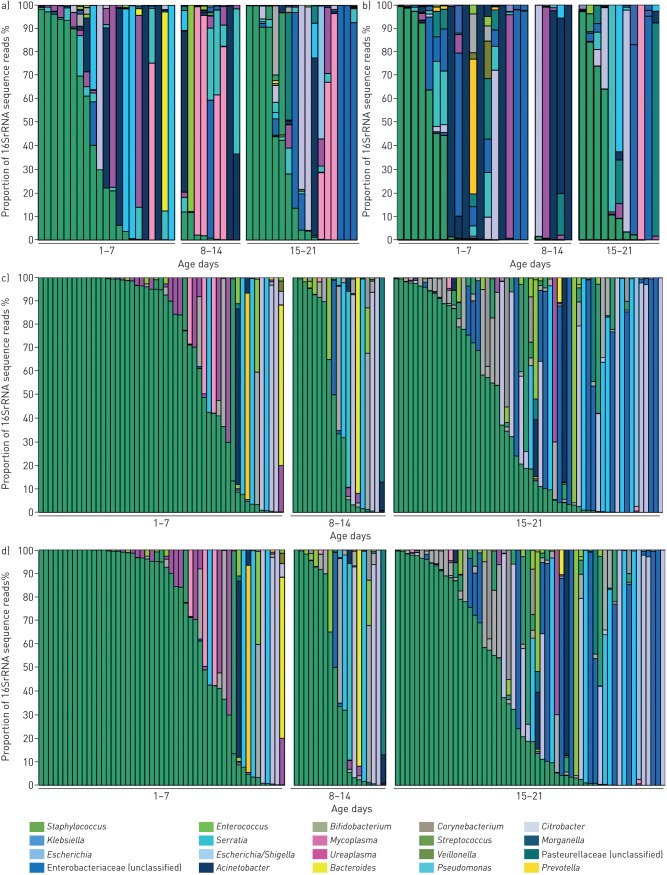
Microbiome analyses for each sample site shown at genus level: a) tracheal aspirate fluid; b) bronchoalveolar lavage; c) nasopharyngeal aspirate; and d) stool. Each bar represents an individual sample with relative abundance shown after all operational taxonomic units from each genus were combined.

The majority of phyla in all samples, as shown in figure 2 and summarised in supplementary figure S1, were Proteobacteria and Firmicutes; Tenericutes were also present, particularly in TAF and BAL samples, and to a lesser extent in NPA samples; Bacteroidetes were noted in stool samples, with lesser presence in NPA, TAF and BAL samples. The pattern of phyla in TAF and BAL samples over the first month of life remained static, with a mean relative abundance ∼40–50% of Proteobacteria, while Proteobacteria in NPA and stool samples increased from a mean relative abundance of 15% in NPA and 30% in stool in the first week of life to 46% and 55%, respectively, by the third/fourth week life (figure 2; summarised data shown in supplementary figure S2). Most samples contained a single dominant phylum (>50% of reads from a single phylum). At genus level, *Staphylococcus*, as shown in figure 3, was the dominant genus (>50% of reads from a single genus) in nearly 30% of all samples, with *Klebsiella*, *Escherichia*, unclassified Enterobacteriaceae, *Enterococcus* and *Serratia* predominating in 35%, and the Tenericutes *Ureaplasma* and *Mycoplasma* for 5.2% of all samples. While Proteobacteria increased and Firmicutes decreased with age, especially in NPA (Proteobacteria p<0.001, Firmicutes p=0.008; Kruskal–Wallis test with Benjamini–Hochberg false discovery rate) and stool samples, there was no obvious pattern of change at genus level, although dominance of the aforementioned genera was evident. Both at genus and OTU level, a dominant genus/OTU was present in most samples, with 31 (91%) out of 34 BAL samples, 43 (92%) out of 47 TAF samples, 122 (97%) out of 126 NPA samples and 130 (90%) out of 145 stool samples having >50% of their reads for a single dominant OTU, as shown in figure 3.

**FIGURE 4:**
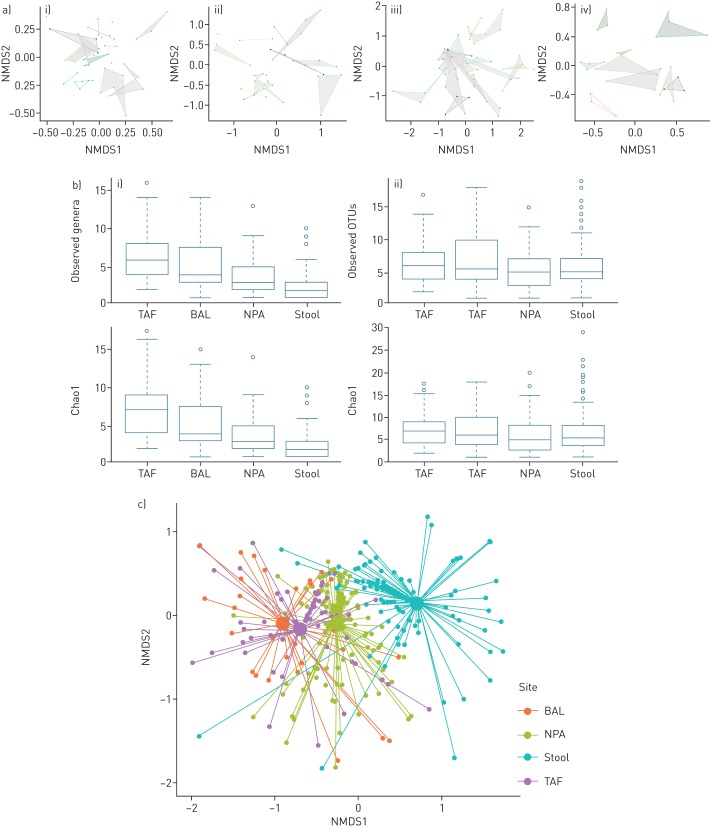
Microbiome community structure between sample sites. a) Nonmetric multidimensional scaling (NMDS) plots for individual babies for each sample site: i) tracheal aspirate fluid (TAF); ii) bronchoalveolar lavage (BAL); iii) nasopharyngeal aspirate (NPA); and iv) stool. Each coloured polygon joins all samples from an individual infant. b) α-diversity of observed genera/operational taxonomic units (OTUs) and Chao1 shown at i) genus and ii) operational taxonomic unit level. c) NMDS plot showing the relationship between the bacterial community between the anatomical sites. PERMANOVA showed significant differences between BAL *versus* TAF, BAL *versus* NPA, BAL *versus* stool, TAF *versus* NPA, TAF *versus* stool and NPA *versus* stool using Bonferroni correction (all p<0.01).

Interestingly, each infant had their own individual bacterial colonisation pattern which differed between individual infants and between anatomical sites (figure 4a and c). Since there was no overall pattern over time, we collated the samples from each anatomical site for further analyses. The number of observed genera and Chao1 index were different between the sample types, being greater in the BAL and TAF samples (p<0.001 for both indices) (figure 4b), but not for Shannon's or Simpson's α-diversity indices (data not shown) when calculated at genus level. However, there were no differences noted for observed OTUs or Chao1 when calculated at OTU level (figure 4b). These data of similar indices for genera and OTU level for BAL and TAF samples suggest that individual OTUs predominate in these samples, but the increase in indices between the genera and OTUs for NPA and stool samples suggest greater richness in these samples. β-diversity analysis for TAF and NPA samples did not show any significant pattern over time (supplementary figure S4).

When bacterial communities were examined between the four sample types by PERMANOVA, all differed significantly from each other, as shown in figure 4c: BAL *versus* TAF; BAL *versus* NPA; BAL *versus* stool; TAF *versus* NPA; TAF *versus* stool; and NPA *versus* stool (all p<0.01).

Delivery mode influences early colonisation across many anatomical sites in term newborn infants and in the limited data for preterm infants in stool samples [7, 25, 26]. Mode of delivery was associated with an increase in Gram-negative genera (*Acinetobacter* (p=0.04) and marginally with *Pseudomonas* (p=0.06)); *Mycoplasma* (p=0.04) in TAF samples; and *Serratia* (p=0.01) in NPA from infants delivered vaginally. For infants delivered by caesarean section, *Staphylococcus* were increased in NPA (p=0.02), and marginally in TAF samples (p=0.056) (supplementary figure S2a). However, sex did not influence TAF or NPA bacterial communities (supplementary figure S2b). A significant difference for bacterial community structure was noted between the recruitment centres (p<0.05) (supplementary figure S2c). This may be partially explained by the outbreak of *Acinetobacter* at one site, resulting in greater *Acinetobacter* observed at that recruitment centre.

At both recruiting centres the initial prophylactic antibiotic regimen of benzylpenicillin and gentamicin was altered according to subsequent microbiological assessments. 219 (79%) out of 276 TAF and 335 (62%) out of 539 NPA samples were taken while the infants were receiving antibiotic therapy (supplementary figure S3). Samples taken when not on antibiotics were significantly more likely to be successfully sequenced for the 16S rRNA gene than samples taken while on antibiotics (OR 2.91, 95% CI 1.48–5.73; p=0.002 for TAF; and 3.10, 2.05–4.67; p<0.0001 for NPA samples). Antibiotics did not affect α-diversity in successfully sequenced TAF and NPA samples, suggesting that they globally suppressed bacterial growth rather than suppressing specific species. Nevertheless, antibiotics were associated with increased proportion of Tenericutes (p=0.03), probably due to *Mycoplasma*, which were marginally increased (p=0.059) in TAF samples compared to the no-antibiotic group.

**FIGURE 5:**
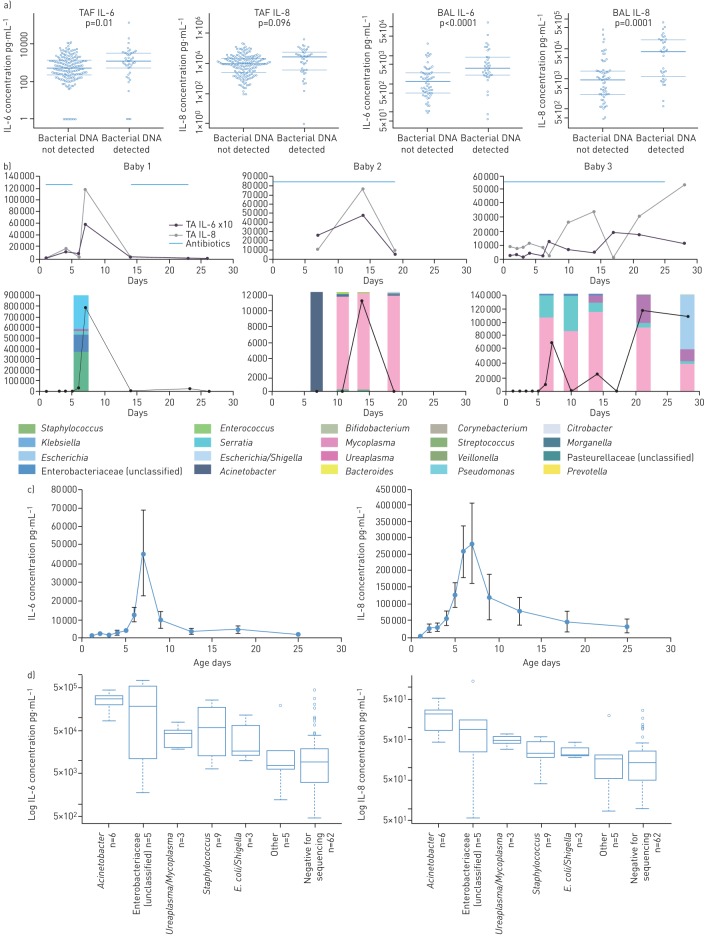
Association of the lower airway microbiome with markers of inflammation. a) Log_10_ scale for interleukin (IL)-6 and -8 in tracheal aspirate fluid (TAF) and bronchoalveolar lavage (BAL) fluid samples for samples where bacterial sequencing was positive or negative. b) The relationship between IL-6, IL-8, antibiotic treatment (blue) in upper graph and bacterial load (black) and bars showing genus constituents in three individual babies. Generally, parallel increases and decreases were observed for all markers despite antibiotic treatment. c) Averaged IL-6 and IL-8 in TAF samples shown for each time point regardless of bacterial presence showing a peak of both cytokines at day 7 as previously shown [2, 3, 19] Data are presented as mean±sem. d) Association between the presence of IL-6 and IL-8 in BAL samples with specific operational taxonomic units (OTUs) showing increased IL-6 and IL-8 if an OTU was identified when compared to the negative for sequencing group. *E. coli*: *Escherichia coli*.

Since a single OTU predominated (>50% of reads) in most of the successfully sequenced samples, potentially those samples with greater initial biomass, we measured IL-6 and IL-8 in all samples to evaluate whether successful bacterial DNA detection in samples was associated with an inflammatory response. IL-6 and IL-8 were largely undetectable in the NPA and faecal fluid extracts, but were noted in considerable quantities in the BAL and TAF samples if they could be sequenced, as shown in figure 5a in TAF and BAL samples: TAF successful sequencing *versus* unsuccessful sequencing samples: IL-6 1222 (534–3224) pg·mL^−1^ *versus* 539 (217–1377) pg·mL^−1^; and IL-8 26 383 (3460–51 740) pg·mL^−1^ *versus* 9031 (2535–20 511) pg·mL^−1^; and BAL IL-6 3629 (2029–8538) pg·mL^−1^ *versus* 1213 (485–2459) pg·mL^−1^; and IL-8 80 689 (12 303–196 477) pg·mL^−1^ *versus* 9520 (3060–18 572) pg·mL^−1^ (all p<0.001). Next, we tracked the dynamic changes in individual infants, as shown in figure 5b. Each individual infant had episodic increases in IL-6 and IL-8 which corresponded to increased bacterial load and to presence of predominant OTUs. From 20 episodic TAF IL-8 peaks of >50 000 pg·mL^−1^ observed in 19 infants, 13 (65%) episodes were associated with presence of bacterial DNA within 24 h of the peak cytokine concentration. Both IL-6 and IL-8 showed peak concentration at 7 days of age as we have shown previously in infants developing CLD [2, 3, 16] (figure 5c). Finally, the predominance of OTUs such as *Acinetobacter*, unclassified Enterobacteriaceae and Mollicutes including *Mycoplasma* and *Ureaplasma* were strongly associated with the greatest increases in IL-6 and IL-8 in BAL, being several-fold greater than in samples which could not be sequenced (figure 5d).

## Discussion

In this comprehensive longitudinal study, we studied the upper and lower airways of preterm infants who were at risk of developing CLD and related the findings to serial stool samples to assess the gut–lung axis. We have made several important observations: first, low bacterial DNA load was noted in the first few days of life which increased during the first month of life. Second, all anatomical sites sampled including the upper and lower airways had their own distinct patterns of bacterial community, in most cases with the presence of a dominant OTU, and with dissimilarity between the anatomical sites. Third, bacterial communities in the lower airways were associated with an active proinflammatory phase. Fourth, specific OTUs were associated with marked increases in proinflammatory cytokines.

Overall, from 1102 samples, 31.9% (n=352) were satisfactorily amplified for bacterial sequencing. Bacterial load was low in all samples during the first 3 days of life, but peaked at days 4–7 of life in BAL and TAF samples, and at days 8–14 in stool and NPA samples. Bacterial acquisition is most likely to be from the mother with vaginally associated microbes including Tenericutes predominating in vaginally delivered infants, and *Staphylococcus* predominating when delivery was by caesarean section.

At phylum level, the NPA and stool samples changed similarly over time with an increase in Proteobacteria, which remained relatively static in the BAL/TAF samples. The Tenericutes, *Ureaplasma* and *Mycoplasma* were mainly confined to the lower airway samples with only some presence in NPA samples, but were almost absent from stool samples. In contrast, *Acinetobacter* was present in stool and in NPA samples, but not in lower airway samples, reflecting the outbreak in that unit. Almost all samples had a predominant OTU, with >90% of samples having a predominant OTU of >50% of reads with *Staphylococcus*, *Mycoplasma* and *Ureaplasma* predominating especially in the lower airways. Interestingly, all infants had their own bacterial pattern with little overlap between the sample types, suggesting an active infective episodic process. Significant differences were present in the community structure of the samples from each anatomical site as was shown by the PERMANOVA analysis. α-diversity indices estimating richness showed greater number of genera present in BAL and TAF than in NPA or stool samples, but the differences disappeared at OTU level suggesting greater richness at OTU level for NPA and stool samples. Since measures of evenness (Shannon diversity index) did not show a difference between the sample sites, the data suggest that the degree of dominance of a single OTU was no different between anatomical sites. Differences between the NPA and BAL/TAF samples may be partly explained by the presence of an uncuffed endotracheal tube which partially separates the upper and lower airways. Obtaining lower airways samples from nonventilated preterm or term newborn infants is not possible, thus limited data exist for the microbial evolution of the lower airways [27].

As previously observed for term-born infants, mode of delivery was associated with increased proportion of *Staphylococcus* in NPA (p=0.022) and marginally in TAF (p=0.056) in those who were delivered by caesarean section and increased *Mycoplasma* (p=0.039) and *Acinetobacter* (p=0.042) in TAF samples and *Serratia* (p=0.008), *Ureaplasma* (p=0.024) and *Corynebacterium* (p=0.028) in NPA samples from vaginally delivered infants. Sex did not affect the bacterial colonisation, but the centre at which the sample was obtained did influence the results. These results may be partly explained by the outbreak of *Acinetobacter* in one unit, but the differences between the two centres remained in sensitivity analyses when the infants colonised with *Acinetobacter* were removed, thus suggesting that the local environment or local personnel appear to influence colonisation in addition to the maternal milieu. Antibiotics, as expected, decreased bacterial load, with fewer samples being suitable for bacterial gene sequencing. However, the diversity and number of OTUs were similar when the babies were on or off antibiotics. Presence of Tenericutes was increased in TAF samples (p=0.03) and a trend towards Tenericutes increased in NPA samples (p=0.059) in the antibiotic group.

Most interestingly, we noted several-fold differences in IL-6 and IL-8 concentrations in BAL and TAF when bacterial sequencing was possible against when it was not. Our data suggest that an infective dysbiotic environment is occurring in the lungs of babies who are at risk of developing CLD. Low biomass samples may have lower cytokine concentrations due to dilution caused by variation in volume of samples returned. However, as per our previous observations, the collated data for IL-6 and IL-8 showed a peak concentration of both cytokines at ∼7 days of age [2, 3, 16]. Increased cytokine peaks associated with predominant OTUs in each individual infant suggest an infective process. This was further supported by the marked increases in TAF IL-6 and IL-8 for individual OTUs confirming that infective processes were common in the lungs of babies who are at risk of developing CLD.

Taken together, these data suggest that both the bacterial community and pulmonary inflammation are potential targets with antimicrobials such as azithromycin, which has both anti-infective and anti-inflammatory activities to decrease rates of CLD [6].

There are several limitations to this study, most notably the presence of an endotracheal tube to support the infant's respiratory system. Most importantly, this precludes the acquisition of samples from “healthy” preterm- and term-born infants which is not ethically possible. The presence of an endotracheal tube may physically separate the lower and upper airways, but, despite prolonged ventilation in many infants, we noted separate processes in the upper and lower airways and in the gastrointestinal tract. The strength of the study is the large number of samples from multiple anatomical sites obtained longitudinally, making this by far the largest study of preterm infants. Furthermore, we have related the findings between recruitment centres and with the inflammatory processes occurring in the lungs of infants at risk of developing CLD.

In summary, bacterial load was low at birth, but quickly increases and microbes are most likely to be acquired from the mother. There was dissimilarity between the upper and lower airways and the gut in bacterial community structures with a separate dysbiotic inflammatory process occurring in the lower airways of infants at increased risk of developing CLD. Additionally, the pulmonary inflammatory processes were occurring despite the infants being on antibiotics suggesting that microbes not susceptible to routinely used antibiotics such as *Ureaplasma* and *Mycoplasma* may predominate and potentially provide a therapeutic target to decrease the development of CLD in preterm-born infants [6].

## Supplementary material

10.1183/13993003.01909-2019.Supp1 **Please note:** supplementary material is not edited by the Editorial Office, and is uploaded as it has been supplied by the author.
Supplementary material ERJ-01909-2019.SUPPLEMENT

## Shareable PDF

10.1183/13993003.01909-2019.Shareable1 This one-page PDF can be shared freely online.
Shareable PDF ERJ-01909-2019.Shareable
