# Supplementary material for: Dissimilarity of the gut–lung axis and dysbiosis of the lower airways in ventilated preterm infants
Source: Eur Respir J. 2020 May 7;55(5):1901909. doi: 10.1183/13993003.01909-2019 (PMC7236867; doi:10.1183/13993003.01909-2019)
Supplement: Supplementary file 1 [file ERJ-01909-2019.SUPPLEMENT.pdf]

Dissimilarity of the gut-lung axis and dysbiosis of the lower airways in ventilated preterm infants – Online Supplement

[1]Gallacher D*, [1]Mitchell E*, [2]Alber D, [3]Wach R, [2]Klein N, [4,5]Marchesi JR, [1]Kotecha S.

[1]Department of Child Health, School of Medicine, Cardiff University, Cardiff, UK.

[2]Institute of Child Health, University College London, London, UK.

[3]Neonatal Unit, North Bristol NHS Trust, Bristol, UK.

[4]School of Biosciences, Cardiff University, Cardiff, UK.

[5]Division of Integrative Systems Medicine and Digestive Disease, Imperial College London, London, UK

*Joint first authors

Address for Correspondence:

Professor Sailesh Kotecha

Department of Child Health

School of Medicine

Cardiff University

Heath Park

Cardiff CF14 4XN

Email: KotechaS@cardiff.ac.uk

Methods

Collection of Clinical Samples

BAL fluid was collected as previously described[1-3]. Briefly, with the infant supine, with the head turned to the left, a Fr6 catheter was advanced through the ET tube until resistance was felt and 1ml/kg of sterile saline instilled. The fluid was then aspirated. After allowing the baby to recover, the procedure was repeated, and the samples pooled. The BAL samples were centrifuged at 10,000g for 10 mins within 60 minutes to separate the cells and supernatant before storage at -80°C.

TA samples were collected at the time of clinically indicated routine endotracheal tube suctioning and the collection catheter flushed with 2ml of sterile saline. Nasopharyngeal aspirates (NPA) samples were collected by suctioning mucous from the nasopharynx and flushing the collected sample with 2 ml of sterile saline. Collected TAF and NPA samples were initially frozen at -20°C then transferred without thawing to -80°C. TA, BAL and NPA samples were obtained daily for the first week of life then twice weekly until 28 days of age or until extubation, whichever occurred earlier. Stool samples were collected twice weekly until 28 days of age. Control samples were obtained flushing sterile saline through the same suction catheters used to collect samples.

DNA Extraction

TAF and NPA samples were thawed on ice and centrifuged at 10,000g for 10 mins to separate the cell pellets and supernatant. Cell pellets from all respiratory samples were resuspended in 0·5ml of DNA extraction buffer containing 5M guarnodine thiocyanate. The samples were bead beaten with 0·1mm zirconia beads for 3 x 30 seconds at 6·5m/s using a FastPrep 24 device (MP Biomedicals, CA, USA) and placed on ice for 5 mins between each episode. Samples were then loaded into a Maxwell 16 automated DNA extraction device (Promega UK, Southampton, UK) using the tissue DNA extraction kit. DNA was eluted into 300ul of the supplied elution buffer. Stool samples were thawed on ice for 1 hour before being weighed before homogenising. The homogenate was then centrifuged, and DNA extracted from the faecal pellet using the Qiagen Stool Mini Kit (Qiagen,

Hildenberg, Germany) as per manufacturer's instructions, with the following optimizations. 2 ml of InhibitEX buffer was added to the faecal pellet and vortexed continuously for 1 minute, or until the sample was fully homogenous. 1·5 ml of this solution was then transferred into a 2 ml MPBiomedicals Lysing Matrix tube E, which contained 1·4 ceramic spheres, 0·1 mm silica spheres and one 4 mm glass bead. The tubes were then homogenised in the FastPrep 24 instrument for 60 seconds at 5·0 m/s. This process was repeated 3 times with 5-minute incubations on ice between each beating. The samples were then heated for 5 minutes at 70°C in a heat block. The heated samples were then vortexed for 15 seconds, before being centrifuged at 20,000 x g for 1 minute. 200 µl of the supernatant was then taken forward into the remainder of the protocol.

Quantification of Bacterial Load

Total bacterial load was quantified by a qPCR assay based on published protocols[4] amplifying the V3-V4 region of the 16S rRNA bacterial gene. Primers sequences used were: forward 5'-CCTACGGGDGGCWGCA-3' and reverse: 5'- GGACTACHVGGGTMTCTAATC -3'. The TaqMan probe sequence was (6FAM) 5'-CAGCAGCCGCGGTA-3' (MGBNFQ). 2·5 µL of DNA was added to a total reaction of 10 µL. The thermal cycling protocol was heating for 3 min at 50°C for UNG treatment, then 10 min at 95°C for Taq activation followed by 40 cycles of 15s at 95°C for denaturation and 1 min at 60°C for annealing and extension. A standard curve was generated in each assay using serially diluted *E. Coli* genomic DNA. DNA used generating standard curves was extracted from pure growths of E.Coli organisms with DNA quantification using a Cubit analyser. Samples, standards and controls were run in triplicate and a set cT threshold maintained for each run of the experiment.

Sequencing

The V3-V4 region of the 16S rRNA gene was amplified from each DNA extract using the Taq Core PCR kit (Qiagen, Hilden, Germany) with forward and reverse primers at 0,5µM each. Barcoded primers were designed according to Kozich *et al.* [5] to include Illumina adapter, an 8 nucleotide barcode

sequence, a 10 nucleotide pad sequence, a 2 nucleotide linker, and a gene-specific primer:: 341F-CCTACGGGNGGCWGCAG or 805R-GACTACHVGGGTATCTAATCC. (Sigma-Aldrich, Dorset, UK) and used to amplify each DNA sample using different barcode combination. The cycling conditions were as follows: initial denaturation at 95^{0}C for 3min, 30 cycles of 95^{0}C for 30 sec, 54^{0}C for 30 sec,72^{0}C for 10 min and a final extension at 72^{0}C for 10 min. Purification of the PCR products was achieved using AMPure beads (Beckman Coulter (UK), High Wycombe, UK).

Purified PCR product were quantified using the Qubit dsDNA High Sensitivity Assay Kit (ThermoFisher, Leicestershire, UK), and pooled to create a library with approximately equal concentrations of 16S rRNA amplicons from each sample. Separate libraries were prepared for TAF, BAL, NPA and stool samples. Pooled libraries were quality and quantity checked using the The High Sensitivity D1000 ScreenTape assay (Agilent Technologies, CA, USA) and a NEBNEXT library quantification kit (New England Biolabs). Each library spiked with 10% PhiX was sequenced on an Illumina MiSeq using the Reagent Kit V2 with 500 cycles (Illumina UK, Cambridge, UK) and custom primers as previously described[5].

Data Analysis

STAMP (Statistical Analysis of Metagenomic Profiles) software was used to produce error bar plots using White's test with Benjamini-Hochberg False Discovery Rate method used to correct for multiple comparisons.

Online Figure 1 – Averaged microbiome data for each sample site shown by phyla

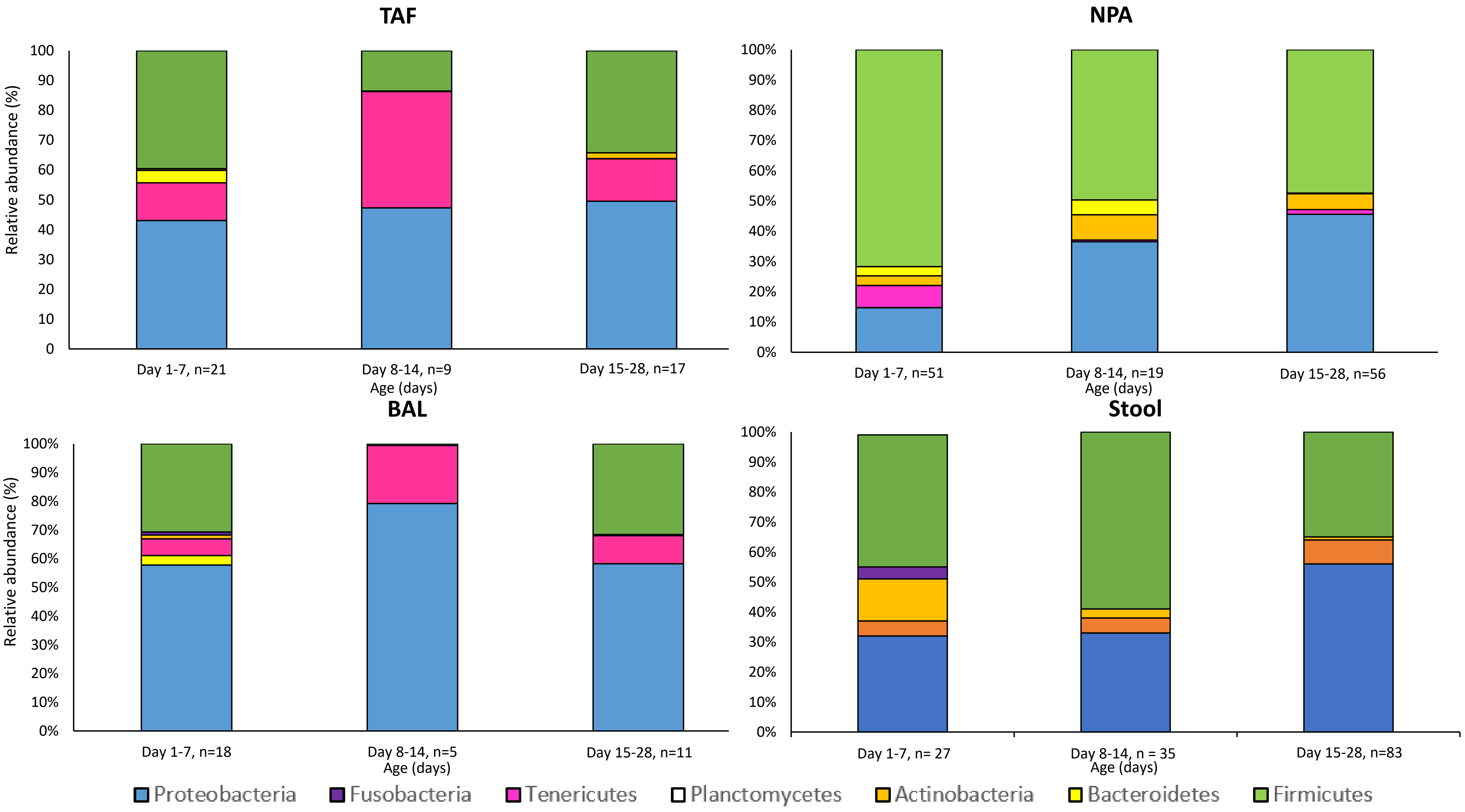

Online Figure 2 – The effect of mode of delivery, gender and centre of recruitment on the lung microbiome

a. Mode of delivery

TAF

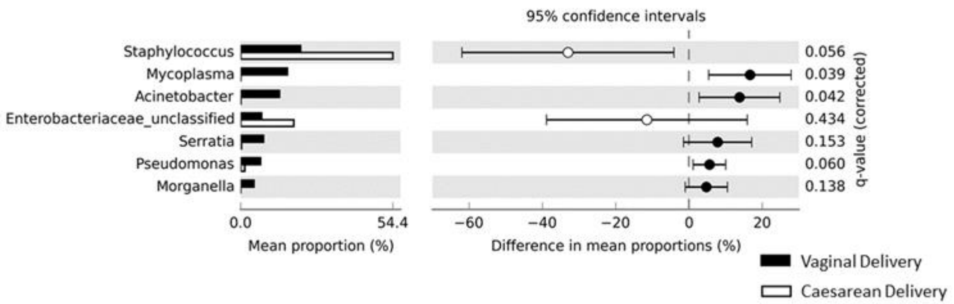

NPA

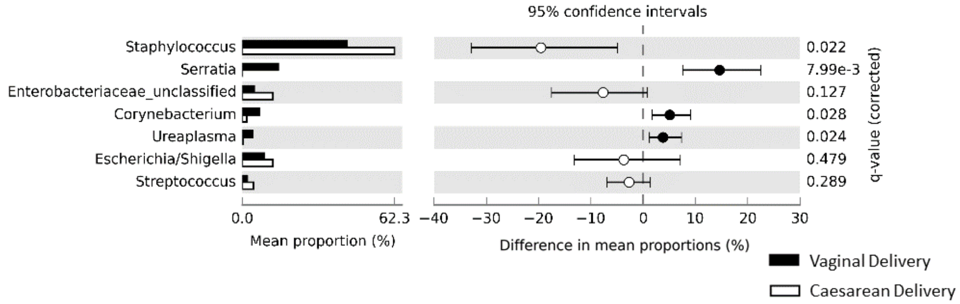

b. Gender

TAF

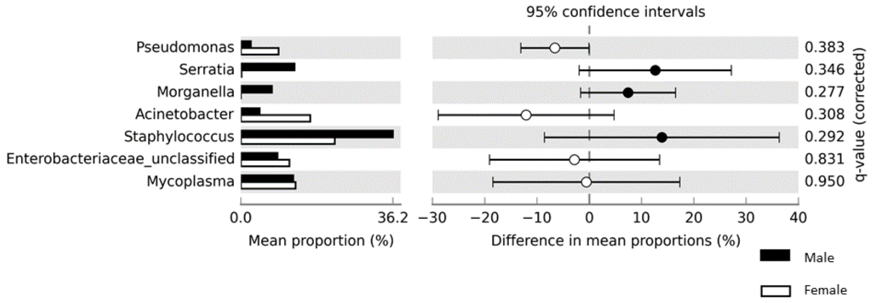

NPA

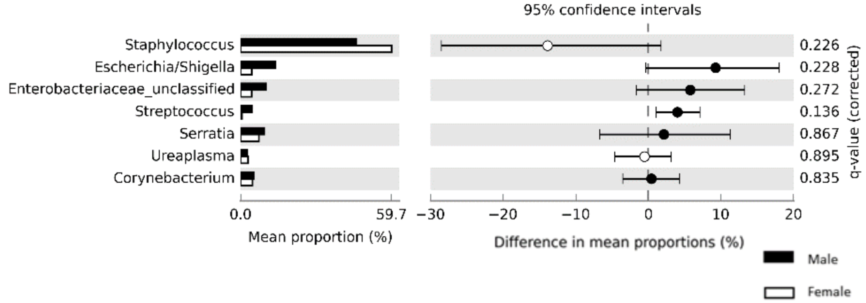

c. Effect of centre of recruitment

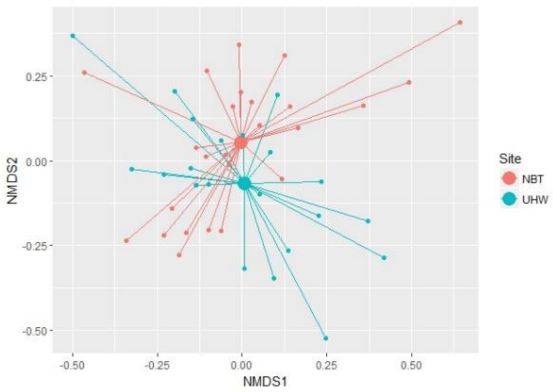

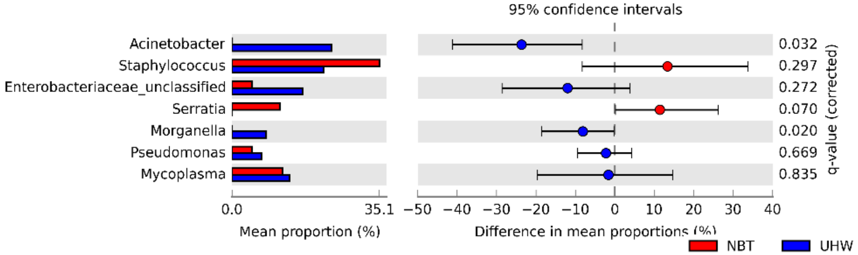

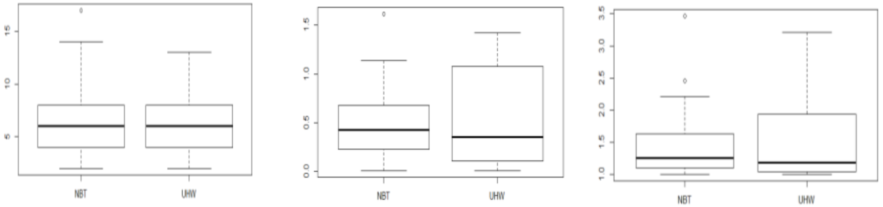

Observed species P=0·673 Shannon P=0·881 Inverse Simpson P=0·966

Online Figure 3 – The effect of antibiotics on tracheal aspirate fluid (TAF) and nasopharyngeal aspiration (NPA)

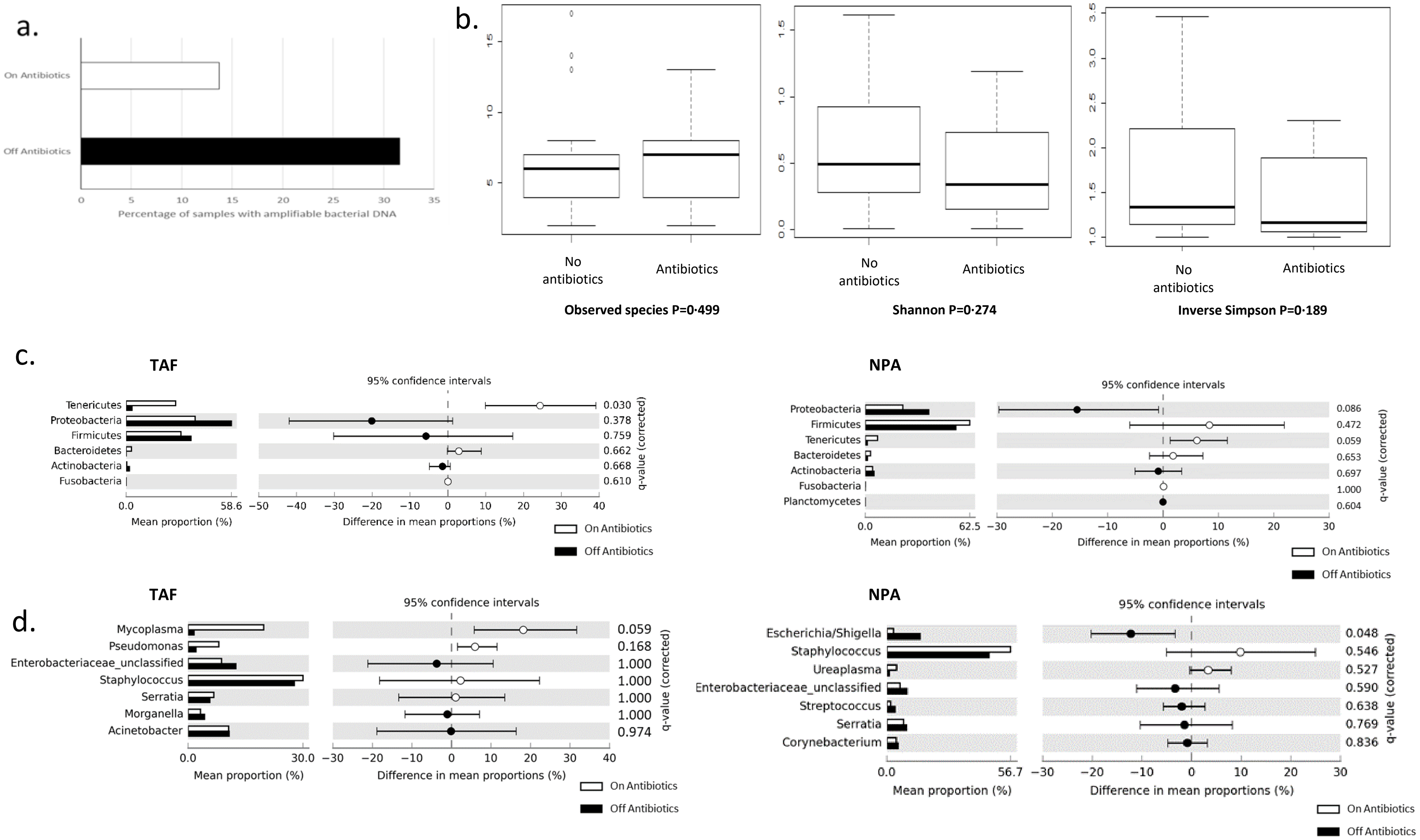

Online Figure 4: Beta-Diversity Changes over time

A - NPA

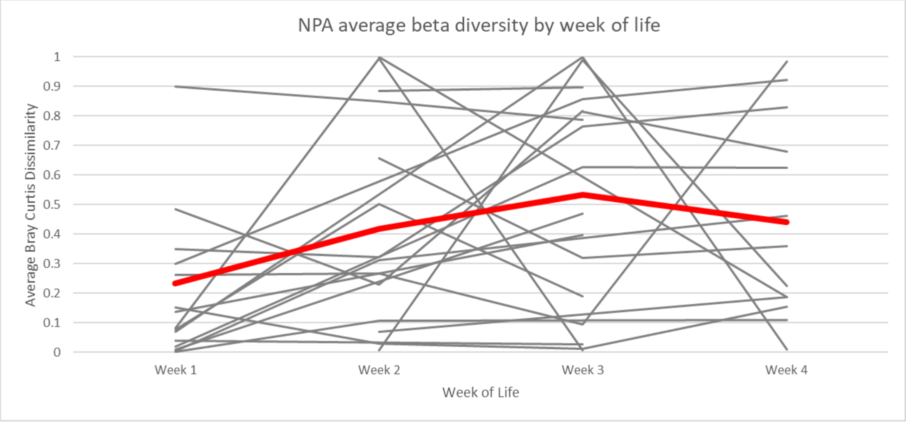

B – TAF

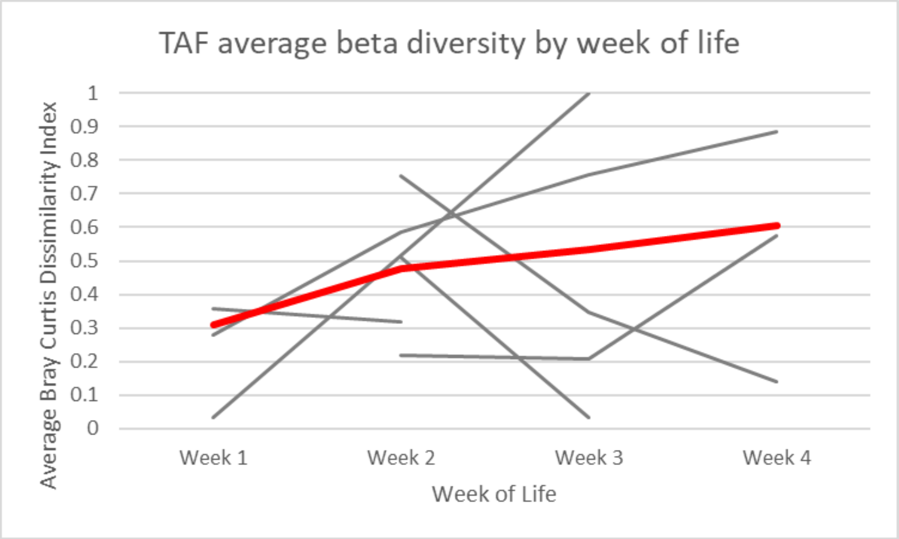

Legends

Online Figure 1. Averaged microbiome data for each sample site shown by phyla. The relative abundance of each phylum averaged for all samples at each time-point for each sample type is shown. Abbreviations: BAL – Bronchoalveolar Lavage, NPA – Nasopharyngeal Aspirate, TAF – Tracheal Aspirate Fluid.

Online Figure 2. The effect of mode of delivery, gender and centre of recruitment on the upper (NPA) and lower airway (TAF) microbiome. Shown are the effect of the (A) mode of delivery, (B) Gender and (C) centre from which sample was collected on bacterial OTUs in upper (NPA) and lower (TAF) airway samples. In addition, alpha and beta diversity shown for sample site (red NBT, North Bristol Trust, UHW, University Hospitals of Wales).

Online Figure 3. The effect of antibiotics on tracheal aspirate fluid (TAF) and nasopharyngeal aspirate (NPA) samples. (A) percentage of samples which had sufficient for bacterial 16s rRNA gene sequencing, whilst on or off antibiotics. (B) Alpha diversity shown for TAF samples when taken when the infant was or was not on antibiotics. (Alpha diversity for NPA similarly similar between the antibiotic and no antibiotic samples.) (C) shows the effect of antibiotics on the lung bacterial community at the phylum level and (D) at genus level, for the most abundant 7 genera, for samples taken whilst on or off antibiotic treatment.

Online Figure 4: Beta-diversity changes over time

Changes in Bray-Curtis Dissimilarity index in samples from individual infants by week of life. Bray-Cutis index calculated between each consecutively collected successfully sequenced sample from each infant. The median day between the samples used to allocate week of life. For weeks with more than 1 beta diversity value calculated the average value was taken. Grey lines represent the

results for individual infants and the bold red line shows the average from each week. A= NPA(Kruskal Wallis p =0.098) , B= TAF(Kruskal Wallis p=0.46).

References

1. Kotecha, S., Wilson, L., Wangoo, A., Silverman, M. & Shaw, R. J. Increase in interleukin (IL)-1 beta and IL-6 in bronchoalveolar lavage fluid obtained from infants with chronic lung disease of prematurity. *Pediatr. Res.* **40,** 250–6 (1996).
2. Beeton, M. L. *et al.* Role of pulmonary infection in the development of chronic lung disease of prematurity. *Eur. Respir. J.* **37,** 1424–30 (2011).
3. de Blic, J. *et al.* Bronchoalveolar lavage in children. ERS Task Force on bronchoalveolar lavage in children. European Respiratory Society. *Eur. Respir. J.* **15,** 217–31 (2000).
4. Liu, C. M. *et al.* BactQuant: An enhanced broad-coverage bacterial quantitative real-time PCR assay. *BMC Microbiol.* **12,** 56 (2012).
5. Kozich, J. J., Westcott, S. L., Baxter, N. T., Highlander, S. K. & Schloss, P. D. Development of a dual-index sequencing strategy and curation pipeline for analyzing amplicon sequence data on the MiSeq Illumina sequencing platform. *Appl. Environ. Microbiol.* **79,** 5112–20 (2013).
