# Supplementary material for: Dissimilarity of the gut–lung axis and dysbiosis of the lower airways in ventilated preterm infants
Source: Eur Respir J. 2020 May 7;55(5):1901909. doi: 10.1183/13993003.01909-2019 (PMC7236867; doi:10.1183/13993003.01909-2019)
Supplement: Supplementary file 2 [file ERJ-01909-2019.Shareable.pdf]

ORIGINAL ARTICLE
PAEDIATRICS

SHAREABLE PDF

Dissimilarity of the gut–lung axis and dysbiosis of the lower airways in ventilated preterm infants

David Gallacher[1,6], Emma Mitchell[1,6], Dagmar Alber[2], Richard Wach[3], Nigel Klein[2], Julian R. Marchesi[4,5] and Sailesh Kotecha[1]

Affiliations: [1]Dept of Child Health, School of Medicine, Cardiff University, Cardiff, UK. [2]Institute of Child Health, University College London, London, UK. [3]Neonatal Unit, North Bristol NHS Trust, Bristol, UK. [4]School of Biosciences, Cardiff University, Cardiff, UK. [5]Division of Integrative Systems Medicine and Digestive Disease, Imperial College London, London, UK. [6]Joint first authors.

Correspondence: Sailesh Kotecha, Dept of Child Health, School of Medicine, Cardiff University, Heath Park, Cardiff CF14 4XN, UK. E-mail: kotechaS@cardiff.ac.uk

@ERSpublications
Respiratory colonisation was acquired after birth and associated with a pro-inflammatory response, suggesting an infectious process was present in babies at risk of developing chronic lung disease of prematurity (CLD), thus providing a target to reduce CLD http://bit.ly/31C27iX

Cite this article as: Gallacher D, Mitchell E, Alber D, *et al*. Dissimilarity of the gut–lung axis and dysbiosis of the lower airways in ventilated preterm infants. *Eur Respir J* 2020; 55: 1901909 [https://doi.org/10.1183/13993003.01909-2019].

This single-page version can be shared freely online.

ABSTRACT
Background: Chronic lung disease of prematurity (CLD), also called bronchopulmonary dysplasia, is a major consequence of preterm birth, but the role of the microbiome in its development remains unclear. Therefore, we assessed the progression of the bacterial community in ventilated preterm infants over time in the upper and lower airways, and assessed the gut–lung axis by comparing bacterial communities in the upper and lower airways with stool findings. Finally, we assessed whether the bacterial communities were associated with lung inflammation to suggest dysbiosis.
Methods: We serially sampled multiple anatomical sites including the upper airway (nasopharyngeal aspirates), lower airways (tracheal aspirate fluid and bronchoalveolar lavage fluid) and the gut (stool) of ventilated preterm-born infants. Bacterial DNA load was measured in all samples and sequenced using the V3–V4 region of the 16S rRNA gene.
Results: From 1102 (539 nasopharyngeal aspirates, 276 tracheal aspirate fluid, 89 bronchoalveolar lavage, 198 stool) samples from 55 preterm infants, 352 (32%) amplified suitably for 16S RNA gene sequencing. Bacterial load was low at birth and quickly increased with time, but was associated with predominant operational taxonomic units (OTUs) in all sample types. There was dissimilarity in bacterial communities between the upper and lower airways and the gut, with a separate dysbiotic inflammatory process occurring in the lower airways of infants. Individual OTUs were associated with increased inflammatory markers.
Conclusions: Taken together, these findings suggest that targeted treatment of the predominant organisms, including those not routinely treated, such as *Ureaplasma* spp., may decrease the development of CLD in preterm-born infants.

Copyright ©ERS 2020. This version is distributed under the terms of the Creative Commons Attribution Licence 4.0.
